# Golem: a flexible and efficient solver for constrained Horn clauses

**DOI:** 10.1007/s10703-025-00470-9

**Published:** 2025-03-26

**Authors:** Martin Blicha, Konstantin Britikov, Natasha Sharygina

**Affiliations:** 1 University of Lugano, Lugano, Switzerland; 2 Charles University, Prague, Czech Republic

**Keywords:** Constrained Horn clauses, Model checking, Satisfiability modulo theories, Software verification

## Abstract

The logical framework of Constrained Horn Clauses (CHC) models verification tasks from a variety of domains, ranging from verification of safety properties in transition systems to modular verification of programs with procedures. In this work we present Golem, a flexible and efficient solver for satisfiability of CHCs over linear real and integer arithmetic. Golem provides flexibility with modular architecture and multiple back-end model-checking algorithms, as well as efficiency with tight integration with the underlying SMT solver. This paper describes the architecture of Golem and its back-end engines, which include our recently introduced model-checking algorithm TPA for deep exploration. The description is complemented by extensive evaluation, demonstrating the competitive nature of the solver.

## Introduction

The framework of *Constrained Horn Clauses* (CHC) has been proposed as a unified, purely logic-based, intermediate format for software verification tasks [1]. CHCs provide a powerful way to model various verification problems, such as safety, termination, and loop invariant computation, across different domains like transition systems, functional programs, procedural programs, concurrent systems, and more [1–4].

The key advantage of CHCs is the separation of modelling from solving, which aligns with the important software design principle—*separation of concerns*. This makes CHCs highly reusable, allowing a specialized CHC solver to be used for different verification tasks across domains and programming languages. The main focus of the front end is then to translate the source code into the language of constraints, while the back end can focus solely on the well-defined formal problem of deciding satisfiability of a CHC system.

CHC-based *verification* is becoming increasingly popular, with several frameworks developed in recent years, including SeaHorn , Korn and TriCera for C [5–7], JayHorn for Java [8], RustHorn for Rust [9], HornDroid for Android [10], SolCMC and SmartACE for Solidity [11, 12]. A novel CHC-based approach for *testing* also shows promising results [13].

The growing demand from verifiers drives the development of specialized *Horn* solvers. Different solvers implement different techniques based on, e.g., model-checking approaches (such as predicate abstraction [14], CEGAR [15] and IC3/PDR [16, 17]), machine learning, automata, or CHC transformations. Eldarica [18] uses predicate abstraction and CEGAR as the core solving algorithm. It leverages Craig interpolation [19] not only to guide the predicate abstraction but also for acceleration [20]. Additionally, it controls the form of the interpolants with *interpolation abstraction* [21, 22]. Spacer [23] is the default algorithm for solving CHCs in Z3 [24]. It extends PDR-style algorithm for nonlinear CHCs [25] with under-approximations and leverages *model-based projection* for predecessor computation. Recently it was enriched with *global guidance* [26]. Ultimate TreeAutomizer [27] implements automata-based approaches to CHC solving [28, 29]. HoIce [30] implements a machine-learning-based technique adapted from the ICE framework developed for discovering inductive invariants of transition systems [31]. FreqHorn [32, 33] combines syntax-guided synthesis [34] with data derived from unrollings of the CHC system.

According to the results of the international competition CHC-COMP [35–37], solvers applying model-checking techniques, namely Spacer and Eldarica, are regularly outperforming the competitors. These are the solvers most often used as the back ends in CHC-based verification projects. However, only specific algorithms have been explored in these tools for CHC solving, limiting their application for diverse verification tasks. Experience from software verification and model checking of transition systems shows that in contrast to the state of affairs in CHC solving, it is possible to build a flexible infrastructure with a unified environment for multiple back-end solving algorithms. CPAchecker [38–43], and Pono [44] are examples of such tools.

This work aims to bring this flexibility to the general domain-independent framework of constrained Horn clauses. We present Golem, a new solver for CHC satisfiability, that provides a unique combination of flexibility and efficiency.^1^ Golem implements several SMT-based model-checking algorithms: our recent model-checking algorithm based on *Transition Power Abstraction* (TPA) [45, 46], and state-of-the-art model-checking algorithms Bounded Model Checking (BMC) [47], *k*-induction [48], Interpolation-based Model Checking (IMC) [49], Lazy Abstractions with Interpolants (LAWI) [50] and Spacer [23]. Golem achieves efficiency through tight integration with the underlying interpolating SMT solver OpenSMT [51, 52] and preprocessing transformations based on *predicate elimination*, *clause merging* and *redundant clause elimination*. The flexible and modular framework of OpenSMT enables customization for different algorithms; its powerful interpolation modules, particularly, offer fine control (in size and strength) with multiple interpolant generation procedures. We report experimentation that confirms the advantage of multiple diverse solving techniques and shows that Golem is competitive with state-of-the-art Horn solvers on large sets of problems.^2^ Overall, Golem can serve as an efficient back end for domain-specific verification tools and as a research tool for prototyping and evaluating SMT- and interpolation-based verification techniques in a unified setting.

An earlier version of this work appeared at CAV 2023 [53]. This article provides a more comprehensive description of Golem ’s internal representation and workings. Additionally, the experiments have been updated to use the most recent version of both Golem and its competitors.

## Tool overview

In this section, we describe the main components and features of the tool together with the details of its usage. For completeness, we recall the terminology related to CHCs first, following the standard literature [2, 54].

### Constrained Horn clauses

Consider a first-order theory $\mathcal {T}$ and a set $\mathcal {R}$ of uninterpreted predicates of fixed arity disjoint from the signature of $\mathcal {T}$. Then a constrained Horn clause is a formula

$$\begin{aligned} \varphi \wedge B_1 \wedge B_2 \wedge \ldots \wedge B_n \implies H \end{aligned}$$

where

- $\varphi$ is an interpreted formula in the language of $\mathcal {T}$,
- each $B_i$ is an application of a relation symbol $p \in \mathcal {R}$ to terms of $\mathcal {T}$,
- *H* is an application of a relation symbol $p \in \mathcal {R}$ to terms of $\mathcal {T}$, or $false$.
- All variables in the formula are implicitly universally quantified.

The antecedent of the implication is commonly denoted as the *body* and the consequent as the *head*. $\varphi$ is referred to as the *constraint*. A clause with head equal to $false$ is commonly called a *query*. A clause with *no* uninterpreted predicate in the body is called a *fact*. When a clause has more than one predicate in the body it is said to be *nonlinear*. A nonlinear system of CHCs has at least one nonlinear clause; otherwise, the system is linear.

The goal of a Horn solver is to prove or disprove satisfiability of the given CHC system. A CHC system is satisfiable if there exists a model $\mathcal {M}$ of $\mathcal {T}$ extended with an interpretation for all the uninterpreted predicates $\mathcal {R}$, such that all the clauses are *valid* in $\mathcal {M}$. This is called *semantic* solvability [54]. In many cases, it is required to express the satisfying interpretation of predicates in the language of the theory $\mathcal {T}$, i.e., the solver needs to provide a mapping of the predicates to the set of formulas in the language of $\mathcal {T}$, $I:\mathcal {R}\rightarrow FLA$, such that each clause from the system is valid in $\mathcal {T}$ after the uninterpreted predicates are replaced by their interpretations. This is called *syntactic* solvability [54]. Note that every system that is syntactically solvable is also semantically solvable, but not the other way around. On the other hand, unsatisfiability of CHCs can be proved by producing a derivation of $false$ from the ground instances of input clauses, using the resolution rule.

### Architecture

**Fig. 1:**
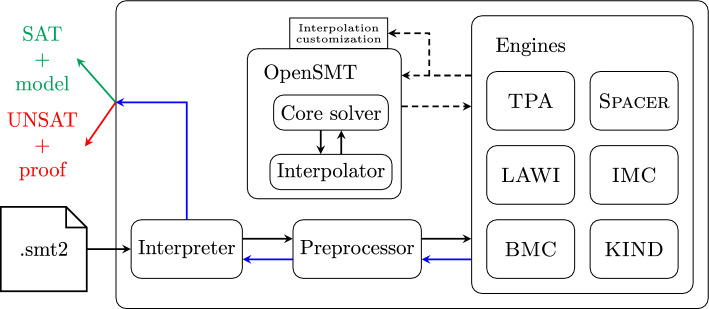
High-level architecture of Golem.

The flow of data inside Golem is depicted in Fig. 1. The system of CHCs is read from .smt2 file, a script in a restricted subset of the SMT-LIB language.^3^Interpreter interprets the SMT-LIB script and builds the internal representation of the system of CHCs. In Golem, CHCs are first *normalized*, then the system is translated into an internal graph representation. Normalization rewrites clauses to ensure that each predicate has only variables as arguments. The graph representation of the system is then passed to the Preprocessor, which applies various transformations to simplify the input graph. Preprocessor then hands the transformed graph to the chosen back-end engine. Engines in Golem implement various SMT-based model-checking algorithms for solving the CHC satisfiability problem. There are currently six engines in Golem: TPA, BMC, KIND, IMC, LAWI, and Spacer (see details in Sec. 3). User selects the engine to run using a command-line option --engine. Golem relies on the interpolating SMT solver OpenSMT [52] not only for answering SMT queries but also for interpolant computation required by most of the engines. Interpolating procedures in OpenSMT can be customized on demand for the specific needs of each engine [55]. Additionally, Golem re-uses the data structures of OpenSMT for representing and manipulating terms.

### Graph representation of clauses

Since its beginning, Golem has been using labeled directed graphs to represent linear CHC systems. To support nonlinear systems, the representation has been generalized to hypergraphs. Intuitively, vertices in the graph correspond to the uninterpreted predicates of the system and edges correspond to the clauses of the system. Additionally, there are two particular vertices $true$ and $false$, where $true$ is understood as the body predicate of *facts*, and $false$ is the head predicate of *queries*. Each edge connects all the body’s predicates to the head’s predicate and is labeled with the *constraint* of the clause represented by the edge.

Formally, given a system of constrained Horn clauses $\mathcal {S}$ over a set of uninterpreted predicates $\mathcal {R}$, the *graph representation* of $\mathcal {S}$ is a labeled directed hypergraph $G = \langle V,E,L \rangle$, where

- $V = \mathcal {R}\cup \{true, false\}$ is the set of vertices,
- $E \subseteq 2^{V} \times V$ is the set of hyperedges corresponding to the clauses of $\mathcal {S}$,
- $L: E \mapsto FLA$ maps each hyperedge to the constraint of the represented clause.

**Fig. 2:**
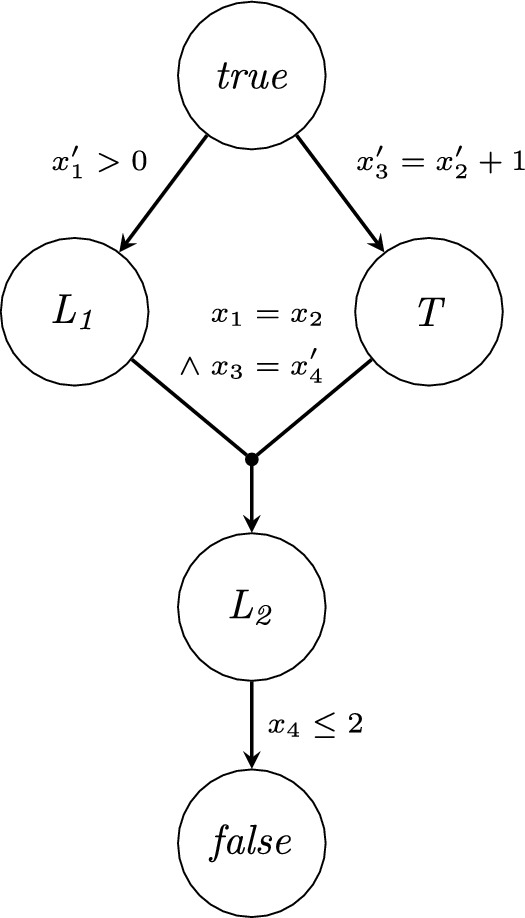
Graph representation of the CHC system from Example 1.

#### Example 1

Consider the following (normalized) CHC system.

1 $$\begin{aligned} x_1^{\prime }> 0&\implies L_1(x_1^{\prime }) \end{aligned}$$

2 $$\begin{aligned} x_3^{\prime } = x_2^{\prime } + 1&\implies T(x_2^{\prime },x_3^{\prime }) \end{aligned}$$

3 $$\begin{aligned} L_1(x_1) \wedge T(x_2,x_3) \wedge x_1= x_2\wedge x_3= x_4^{\prime }&\implies L_2(x_4^{\prime }) \end{aligned}$$

4 $$\begin{aligned} L_2(x_4) \wedge x_4\le 2&\implies false \end{aligned}$$

Clauses (1) and (2) are facts, clause (4) is a query. Clause (3) is nonlinear, because it has two uninterpreted predicates, $L_1$ and *T* in the body. The graph representation of this system is depicted in Fig. 2.

Graph representation of the clauses is suitable both for applying preprocessing transformations and as an input to the back-end engines. In preprocessing, many transformations from one CHC system to a equisatisfiable one can be expressed as familiar graph operations, such as merging edges or contracting vertices (see Sect. 2.5 for details). In the back-end engines, deciding (un)satisfiability of a CHC system is translated to a familiar problem of reachability in the graph, i.e., deciding existence of a feasible path from $true$ to $false$.

### Models and proofs

Besides solving the CHC satisfiability problem, a *witness* for the answer is often required by the domain-specific application. Satisfiability witness is a *model*, an interpretation of the CHC predicates that makes all clauses valid. Unsatisfiability witness is a *proof*, a derivation of the empty clause from the input clauses. In software verification these witnesses correspond to program invariants and counterexample paths, respectively. All engines in Golem produce witnesses for their answer. Witnesses from engines are translated back through the applied preprocessing transformations. Only after this *backtranslation*, the witness matches the original input system and is reported to the user. Witnesses must be explicitly requested with the option --print-witness.

When reporting an unsatisfiability proof to the user, Golem follows the trace format proposed by Eldarica . Internally, proofs are stored as a sequence of derivation steps. Every derivation step represents a ground instance of a clause from the system. The ground instances of predicates from the body form the *premises* of the step, and the ground instance of the head’s predicate forms the *conclusion* of the step. For the derivation to be valid, the premises of each step must have been derived earlier, i.e., each premise must be a conclusion of some derivation step earlier in the sequence. To the user, the proof is presented as a sequence of derivations of ground instances of the predicates, where each step is annotated with the indices of its premises. Example 2 illustrates the proof format.

#### Example 2

The CHC system from Example 1 is unsatisfiable and the following is proof of its unsatisfiability, as reported by Golem.

$$\begin{aligned} 1.\ &L_1(1) & \\ 2.\ &T(1,2) & \\ 3.\ &L_2(2) & ;1,2\\ 4.\ &false & ;3 \end{aligned}$$

The derivation of $false$ consists of four steps. Step 1 instantiates the first clause for $x_1^{\prime }:= 1$. Step 2 instantiates the second clause for $x_2^{\prime }:= 1$ and $x_3^{\prime }:= 2$. Step 3 applies resolution to the instance of the third clause for $x_1:= 1$, $x_2:= 1$, $x_3:= 2$ and $x_4^{\prime }:= 2$ and facts derived in steps 1 and 2. Finally, step 4 applies resolution to the instance of the fourth clause for $x_4:= 2$ and the fact derived in step 3.

Witnesses of satisfiability, i.e, models, are internally stored as formulas in the background theory, using only the variables of the (normalized) uninterpreted predicates. They are presented to the user in the format defined by SMT-LIB [56]: a sequence of SMT-LIB’s define-fun commands, one for each uninterpreted predicate.

#### Example 3

Consider the CHC system from Example 1, but with clause (4) replaced with the following clause with a stronger constraint.

$$\begin{aligned} \qquad \qquad \qquad \qquad \qquad \qquad \qquad \qquad L_2(x_4) \wedge x_4< 2 \implies false \quad \quad \quad \quad \qquad \qquad \qquad \qquad \qquad \qquad ({4^\prime }) \end{aligned}$$

This modified system is satisfiable and Golem finds the following interpretation for the predicates $L_1, L_2, T$:

$$\begin{aligned} L_1(x_1)&\equiv (x_1 \ge 1) \\ T(x_2, x_3)&\equiv ( x_2 - x_3 \ge 1) \\ L_2(x_4)&\equiv (x_4 \ge 2) \\ \end{aligned}$$

The actual output from Golem, following the SMT-LIB format, is

$$\begin{aligned}\begin{array}{lll}(\text{define}-\text{fun}\, \text{L}1\,\, ((\text{x}1\, \text{Int}))\,\,\, \text{Bool}\,\,(<=\, 1\,\, \text{x1}))\\(\text{define}-\text{fun}\,\text{T}\,\, ((\text{x}2\, \text{Int})\,\,(\text{x}3\, \text{Int}))\,\,\, \text{Bool}\,\,(<=\, 1\, \,\,(-\text{x}3\,\, \text{x2})))\\ (\text{define}-\text{fun}\, \text{L}2\,\, ((\text{x4}\,\, \text{Int}))\,\,\, \text{Bool}\,\,(<=\, 2\,\,\, \text{x4}))\end{array}\end{aligned}$$

In addition to witness production, Golem implements an internal *validator* that checks the correctness of produced witnesses. Models are checked by substituting each uninterpreted predicate by its interpretation from the model and checking logical validity of all the clauses with OpenSMT. Proofs are validated by checking correctness of every derivation step. Each derivation step corresponds to a ground instance of some clause and the check of correctness must verify that the step’s premises correspond to the predicates in the body of the clause and that the constraint of the ground instance is satisfied. As implemented, Golem only remembers the derived ground instances of the predicates and reconstructs the ground instance of the clause on demand. Validation in Golem is enabled with an option --validate and serves primarily as a debugging tool for the developers of witness production.

### Preprocessing transformations

Preprocessing can significantly improve performance by transforming the input CHC system into one more suitable for the back-end engine. Golem implements a few transformations, all of which operate on the graph representation. We describe the transformations implemented in Golem in detail below.

An important feature of Golem is that all applied transformations are *reversible* in the sense that any model or proof for the transformed system can be translated back to a model or proof of the original system.

*Predicate elimination* Assuming a given predicate is not present in both the body and the head of the same clause, this predicate can be eliminated by exhaustive application of the resolution rule. In terms of our graph representation, this corresponds to vertex contraction. In Golem, vertex contraction is currently limited to vertices appearing only in simple edges (i.e., edges having only a single source), not hyperedges. Contraction of a vertex *v* means that *v* and all its edges are removed from the graph, and a new edge is added to the graph for every pair of edges (*s*, *v*) and (*v*, *t*). The label of the new edge is a *conjunction* of the labels of the two replaced edges, with appropriate renaming of the variables. Note that while this transformation reduces the number of vertices in the graph, it replaces *n* incoming and *m* outgoing edges with $n \times m$ new edges. To avoid blowup in the number of edges, contraction can be limited to the case when either *n* or *m* is 1.

*Clause merging* Clause merging is a transformation that merges all clauses with the same uninterpreted predicates in the body and the head to a single clause by disjoining their constraints. In the graph representation, this replaces all edges with the same sources and target by a single edge labeled by the disjunction of the labels of replaced edges. This transformation effectively pushes work from the level of the model-checking algorithm in the back-end engine to the underlying level of the SMT solver.

*Redundant clause detection* The input CHC system can contain clauses which can never participate in the proof of unsatisfiability. Such clauses can be safely removed from the system without affecting its satisfiability. Golem detects these clauses by traversing the graph backward from the vertex $false$ and marking reachable vertices. If a vertex has not been seen, it cannot be a part of any path from $true$ to $false$. Thus, such a vertex can be safely deleted from the graph, together with all its incoming and outgoing edges.

*Constraint simplification* Constraints in a CHC system can be replaced by equivalent formulas without affecting the satisfiability of the system. Golem applies standard normalizations and simplifications of terms in constraints. Additionally, Golem attempts to eliminate auxiliary variables from the constraint, i.e., variables that do not occur in any of the clause’s uninterpreted predicates. If $\varphi$ is of the form $x = t \wedge \varphi '(x)$ then it can be simplified to $\varphi '(t)$.

## Back-end engines of Golem

The core components of Golem that solve the problem of satisfiability of a CHC system are referred to as *back-end engines*, or just engines. Golem implements several popular state-of-the-art algorithms from model checking and software verification: BMC [47], *k*-induction [48], IMC [49], LAWI [50] and Spacer [23]. These algorithms treat the problem of solving a CHC system as a *reachability* problem in the graph representation.

The unique feature of Golem is the implementation of the new model-checking algorithm based on the concept of *Transition Power Abstraction* (TPA). It is capable of much deeper analysis than other algorithms when searching for counterexamples [45], and it discovers *transition* invariants [46], as opposed to the usual (state) invariants.

### Transition power abstraction

The TPA engine in Golem implements the model-checking algorithm based on the concept of Transition Power Abstraction. It can work in two modes: The first mode implements the basic TPA algorithm, which uses a single TPA sequence [45]. The second mode implements the more advanced version, split-TPA, which relies on two TPA sequences obtained by splitting the single TPA sequence of the basic version [46]. In Golem, both variants use the under-approximating *model-based projection* for propagating truly reachable states, avoiding full quantifier elimination. Moreover, they benefit from incremental solving available in OpenSMT, which speeds up the satisfiability queries.

The TPA algorithms, as described in the publications, operate on transition systems [45, 46]. However, the engine in Golem is not limited to a single transition system. It can analyze a connected *chain of transition systems*. In the software domain, this model represents programs with a sequence of consecutive loops. The extension to the chain of transition systems works by maintaining a separate TPA sequence for each node on the chain, where each node has its own transition relation. The reachable states are propagated forwards on the chain, while safe states—from which final error states are unreachable—are propagated backwards. In this scenario, transition systems on the chain are queried for reachability between various initial and error states. Since the transition relations remain the same, the summarized information stored in the TPA sequences can be re-used across multiple reachability queries. The learnt information summarizing multiple steps of the transition relation is not invalidated when the initial or error states change.

Golem’s TPA engine discovers counterexample paths in unsafe transition systems, which readily translate to unsatisfiability proofs for the corresponding CHC systems. For safe transition systems, it discovers safe *k*-inductive transition invariants. If a model for the corresponding CHC system is required, the engine first computes a quantified inductive invariant and then applies quantifier elimination to produce a quantifier-free inductive invariant, which is output as the corresponding model.

The TPA engine’s ability to discover deep counterexamples and transition invariants gives Golem a unique edge for systems requiring deep exploration.

### Engines for state-of-the-art model-checking algorithms

Besides TPA, Golem implements several popular state-of-the-art model-checking algorithms.

*Bounded model checking,* *k*-*induction, interpolation-based model checking* Standard algorithms BMC [47], *k*-induction [48] and IMC [49] originated in hardware model checking and operate on transition systems defined by (symbolically represented) initial states $Init$, transition relation $Tr$, and error states $Bad$. Their implementation in Golem internally translate CHC representation of a transition system to this standard representation and then faithfully follows the description of the algorithms in the respective publications. All three algorithms check existence of a path of gradually increasing lengths between the initial and the errors states. Formally, they check the satisfiability of the formula

$$\begin{aligned} Init (x^{(0)}) \wedge \bigwedge _{i=0}^{n-1} Tr (x^{(i)},x^{(i+1)}) \wedge Bad (x^{(n)}) \end{aligned}$$

for increasing values of *n*. If this formula is satisfiable for some value of *n*, a counterexample path of length *n* has been found. While BMC focuses only on search for counterexample paths, *k*-induction and IMC also attempt to prove *absence* of counterexamples.

*k*-induction complements the counterexample checks with checks if the negation of error states (the safety property) is *k*-inductive. This amount to checking if

$$\begin{aligned} \bigwedge _{i=0}^{k-1}(\lnot Bad (x^{(i)}) \wedge Tr (x^{(i)},x^{(i+1)})) \implies \lnot Bad (x^{(k)}) \end{aligned}$$

is logically valid, i.e., its negation is unsatisfiable. If that is the case, and at the same time no counterexample up to length *k* has been found, then the safety property is a *k*-inductive invariant of the system and the error states can never be reached.

Interpolation-based model checking takes a different approach. It uses Craig interpolation [19] to over-approximate the set of safe reachable states. If such an approximation is shown to be inductive, i.e., the system cannot escape from this set with one step of the transition relation, then the error states can never be reached.

*Lazy abstraction with interpolants* *Lazy Abstractions with Interpolants* (LAWI) is an algorithm introduced by McMillan for verification of software [50].^4^ It operates in the framework of lazy abstraction [57] and counterexample-guided abstraction refinement (CEGAR) [15]. Interpolants, computed from refutations of spurious counterexample paths, guide the refinement process. As introduced by McMillan, the algorithm operates on programs represented with *abstract reachability graphs*. These map straightforwardly to *linear* CHC systems, which is the input supported by our implementation of the algorithm in Golem.

S**pacer** Spacer engine in Golem implements the IC3-based algorithm originally named Rec and implemented in the tool Spacer [23, 58]. Original Spacer is now the default fixed-point engine and Horn solver in Z3 [24]. Z3-S*pacer* has been extended several times with various optimizations and support for theories beyond arithmetic since the original publication [26, 59–61].

The implementation in Golem follows the description from the journal publication [23]. Spacer algorithm heavily relies on efficient approximations for quantifier elimination. Typically, Craig interpolation is used to *over-approximate* quantifier elimination and *model-based projection* (MBP) [23] is used to *under-approximate* it. Golem relies on OpenSMT for interpolation but implements its own MBP procedure for integer and real linear arithmetic, based on another description of the procedure [62].

The advantage of the Spacer algorithm is that it works over any CHC system, even nonlinear ones. Nonlinear CHC systems can model programs with summaries, and in this setting, Spacer computes both under-approximating and over-approximating summaries of the procedures to achieve modular analysis of programs. Spacer is currently the only engine in Golem capable of solving nonlinear CHC systems.

### Requirements on the underlying SMT solver

All engines in Golem rely on OpenSMT for answering SMT queries, often leveraging the incremental capabilities of OpenSMT. Efficient incremental satisfiability solving is crucial to the performance of BMC, KIND, Spacer, and TPA engines. While BMC and KIND issue relatively few hard SMT queries, TPA and Spacer, on the other hand, typically issue large number of relatively simple queries. Additionally, the engines IMC, LAWI, Spacer and TPA heavily use the flexible and controllable interpolation framework in OpenSMT [55, 63], especially multiple interpolation procedures for linear-arithmetic conflicts [64, 65]. While in most cases binary interpolation is sufficient, LAWI actually relies on an efficient computation of *path interpolants* [66, 67] from a single refutation proof.

## Experiments

In this section, we evaluate the performance of Golem ’s individual engines on the benchmarks from CHC-COMP 2022. The goal of these experiments is to (1) demonstrate the usefulness of multiple back-end engines and their potential combined use for solving various problems, and (2) compare Golem against state-of-the-art Horn solvers. The benchmark collections of CHC-COMP represent a rich source of problems from various domains.^5^ Version 0.5.0 of Golem was used for these experiments. Z3-Spacer (Z3 4.13.0) and Eldarica 2.0.9 were run (with default options) for comparison as the best Horn solvers available. Earlier version of these experiments [53] used older releases of these tools. Compared to these older releases, Golem’s performance has improved, especially its Spacer and LAWI engines; Eldarica’s performance stayed the same; Z3-Spacer has significantly improved on satisfiable benchmarks in the category LIA-nonlin, but its performance has worsened on unsatisfiable benchmarks from the same category and also on the benchmarks from the category LIA-lin. Overall trends have not changed significantly.

All experiments were conducted on a machine with an AMD EPYC 7452 32-core processor and 8x32 GiB of memory; the timeout was set to 300 s. No conflicting answers were observed in any of the experiments. The results are in line with the results of the last editions of CHC-COMP where Golem participated [36, 37]. Our artifact for reproducing the experiments is available at https://doi.org/10.5281/zenodo.10900551.

### Category LRA-TS

We ran all engines of Golem on all 498 benchmarks from the LRA-TS (transition systems over linear real arithmetic) category of CHC-COMP.

**Table 1:** Number of solved benchmarks from LRA-TS category

	BMC	KIND	IMC	LAWI	Spacer	split-TPA	VB
SAT	0	260	140	288	214	128	364
UNSAT	86	85	70	76	70	72	86

**Fig. 3:**
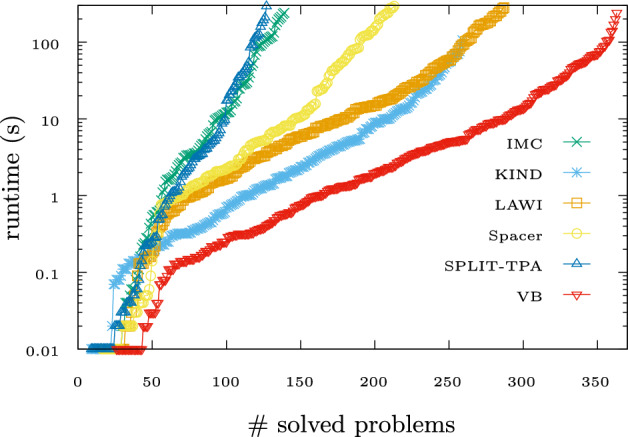
Performance of Golem’s engines on SAT problems of LRA-TS category.

Table 1 shows the number of benchmarks solved per engine, together with a *virtual best* (VB) engine.^6^ On unsatisfiable problems, the differences between the engines’ performance are not substantial, but the BMC engine firmly dominates the others. On satisfiable problems, we see significant differences. Figure 3 plots, for each engine, the number of solved *satisfiable* benchmarks (x-axis) within the given time limit (y-axis, log scale).

The large lead of VB suggests that the solving abilities of the engines are widely complementary. No single engine dominates the others on satisfiable instances. The *portfolio* of techniques available in Golem is much stronger than any single one of them.

Moreover, the unified setting enables direct comparison of the algorithms. For example, we can conclude from these experiments that the extra check for *k*-inductive invariants on top of the BMC-style search for counterexamples, as implemented in the KIND engine, incurs only a small overhead on unsatisfiable problems, but makes the KIND engine very successful in solving satisfiable problems.

### Category LIA-Lin

Next, we considered the LIA-Lin category of CHC-COMP. These are linear systems of CHCs with linear integer arithmetic as the background theory. There are many benchmarks in this category, and for the evaluation at the competition, a subset of benchmarks is selected (see [36, 37]). We evaluated the LAWI and Spacer engines of Golem (the engines capable of solving general linear CHC systems) on the benchmarks selected at CHC-COMP 2022 and compared their performance to Z3-Spacer and Eldarica. Notably, we also examined a specific subcategory of LIA-lin, namely extra-small-lia^7^ with benchmarks that fall into the fragment accepted by Golem ’s TPA engine.

There are 55 benchmarks in extra-small-lia subcategory, all satisfiable, but known to be highly challenging for all tools. The results, given in Table 2, show that split-TPA outperforms not only LAWI and Spacer engines in Golem, but also Z3-S*pacer*. Only Eldarica solves more benchmarks. We ascribe this to split-TPA’s capability to perform deep analysis and discover transition invariants.

**Table 2:** Number of solved benchmarks from extra-small-lia subcategory

Golem				
split-TPA	LAWI	Spacer	Z3-S*pacer*	Eldarica
23	13	18	17	36

For the whole LIA-Lin category, 499 benchmarks were selected in the 2022 edition of CHC-COMP [37]. The perfo Golemrmance of the LAWI and Spacer engines of , Z3-Spacer and Eldarica on this selection is summarized in Table 3. Here, the Spacer engine of Golem significantly outperforms the LAWI engine. Moreover, even though Golem loses to Z3-Spacer, it beats Eldarica. Given that Golem is a prototype, and Z3-Spacer and Eldarica have been developed and optimized for several years, this demonstrates the great potential of Golem.

**Table 3:** Number of solved benchmarks from LIA-Lin category

	Golem			
	LAWI	Spacer	Z3-Spacer	Eldarica
SAT	151	189	198	182
UNSAT	86	84	89	60

### Category LIA-Nonlin

**Fig. 4:**
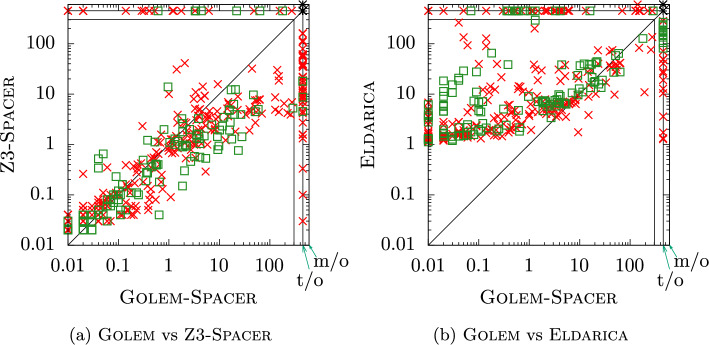
Comparison on LIA-Nonlin category (red cross - SAT, green box - UNSAT).

Finally, we considered the LIA-Nonlin category of benchmarks of CHC-COMP, which consists of *nonlinear* systems of CHCs with linear integer arithmetic as the background theory. For the experiments, we used the 456 benchmarks selected for the 2022 edition of CHC-COMP. Spacer is the only engine in Golem capable of solving nonlinear CHC systems; thus, we focused on a more detailed comparison of its performance against Z3-Spacer and Eldarica. The results of the experiments are summarized in Fig. 4 and Table 4.

**Table 4:** Number of solved benchmarks from LIA-Nonlin category. The number of *uniquely* solved benchmarks is in parentheses

	Golem-S*pacer*	Z3-Spacer	Eldarica
SAT	241 (6)	267 (36)	218 (6)
UNSAT	126 (2)	123 (1)	122 (8)

Overall, Golem solved fewer problems than Z3-Spacer but more than Eldarica; however, *all* tools solved some instances *uniquely*. A detailed comparison is depicted in Fig. 4. For each benchmark, its data point in the plot reflects the runtime of Golem (x-axis) and the runtime of the competitor (y-axis). The plots suggest that the performance of Golem is often orthogonal to Eldarica, but highly correlated with the performance of Z3-Spacer. This is not surprising as the Spacer engine in Golem is built on the same core algorithm. Even though Golem is often slower than Z3-Spacer, there is a non-trivial amount of benchmarks on which Z3-Spacer times out, but which Golem solves fairly quickly. Thus, Golem, while being a newcomer, already complements existing state-of-the-art tools, and more improvements are expected in the near future.

To summarize, the overall experimentation with different engines of Golem demonstrates the advantages of the multi-engine general framework and illustrates the competitiveness of its analysis. It provides a lot of flexibility in addressing various verification problems while being easily customizable with respect to the analysis demands.

## Conclusion

In this work, we presented Golem, a flexible and efficient Horn solver with multiple back-end engines, including recently-introduced TPA-based model-checking algorithms. Golem is a suitable research tool for prototyping new SMT-based model-checking algorithms and comparing algorithms in a unified framework. Additionally, the effective implementation of the algorithms achieved with tight coupling with the underlying SMT solver makes it an efficient back end for domain-specific verification tools. Future directions for Golem include support for VMT input format [68] and analysis of liveness properties, extension of TPA to nonlinear CHC systems, implementation of more back-end algorithms such as PD-KIND [69] and HoIce [30], and support for SMT theories of arrays, bit-vectors and algebraic datatypes.

## Data Availability

An artifact for reproducing our experiments, together with all benchmarks and Golem’s source code is available at https://doi.org/10.5281/zenodo.10900551. Golem is also available at https://github.com/usi-verification-and-security/golem.

## Footnotes

1 Golem is available at https://github.com/usi-verification-and-security/golem.

2 This is in line with results from CHC-COMP 2021 and 2022 [36, 37]. In 2022, Golem beat all other solvers except Z3-Spacer in the LRA-TS, LIA-Lin and LIA-Nonlin tracks.

3 https://chc-comp.github.io/format.html.

4 It is also known as Impact, which was the first tool that implemented the algorithm.

5 https://github.com/orgs/chc-comp/repositories.

6 Virtual best engine picks the best performance from all engines for each benchmark.

7 https://github.com/chc-comp/extra-small-lia.
